# Supplementary material for: Inhibition of Escherichia Virus MS2, Surrogate of SARS-CoV-2, via Essential Oils-Loaded Electrospun Fibrous Mats: Increasing the Multifunctionality of Antivirus Protection Masks
Source: Pharmaceutics. 2022 Jan 27;14(2):303. doi: 10.3390/pharmaceutics14020303 (PMC8875402; doi:10.3390/pharmaceutics14020303)
Supplement: Supplementary file 1 [file pharmaceutics-14-00303-s001.zip › pharmaceutics-1544233-supplementary.pdf]

pharmaceutics

MDPI

Supplementary Materials: Inhibition of *Escherichia* virus MS2, Surrogate of SARS-CoV-2, Via Essential Oils-Loaded Electrospun Fibrous Mats: Increasing the Multifunctionality of Antivirus Protection Masks

Joana M. Domingues, Marta O. Teixeira, Marta A. Teixeira, David Freitas, Samira F. da Silva, Shafagh D. Tohidi, Rui D. V. Fernandes, Jorge Padrão, Andrea Zille, Carla Silva, Joana C. Antunes and Helena P. Felgueiras

Evaluator Nº________ Age: ________ Gender: M/F Smell Problems: Y/N Smoker: Y/N

Were you ever infected with Covid-19: Y/N
Diagnosis Date:______________ Cure date:______________

This study intends to conduct an olfactory analysis of polymer mats with/without incorporated essential oils. To complete the questionnaire, please smell all samples (sequentially, each sample once) by holding the sample close to the nose (≈ 1 to 2 cm from the nose) for 30 sec. A 30 sec interval between samples should be respected, continuing the measurements after this interval. Then, please answer to questions 1-4.

Samples: A B C D E F G

1. Recognition of differences in intensity
Please complete the following table by placing the samples' code in the blank spaces, in ascending order of intensity.
Odor intensity

Sample							

Low → High

2. Intensity evaluation
Please establish an odor intensity correlation by writing the samples' code on the right column of the table, as you see fit.

Odor intensity	Sample(s)
0 = not perceptible	
1 = weakly/not perceptible	
2 = moderately perceptible	
3 = clearly perceptible	
4 = strongly perceptible	
5 = very strongly perceptible	

3. Intensity between two samples
Please select (by placing an X in the corresponding blank space) the sample with the highest intensity in each pair.
Odor intensity

Sample B	Sample C

Sample D	Sample E

Sample F	Sample G

4. Affective evaluation
Please establish an affective evaluation correlation by writing the samples' code on the right column of the table, as you see fit.

Affective evaluation	Sample(s)
-4 = extremely unpleasant	
-2 = unpleasant	
0 = not unpleasant, not pleasant	
+2 = pleasant	
+4 = extremely pleasant	

Figure S1. Sensory evaluation questionnaire.

Table S1. Solid-phase microextraction followed by gas chromatography-mass spectrometry method (SPME-GC-MS) peak identification and quantification in LGO (examined at concentration of 1 mg/mL).

Entry	Retention Time (min)	Compound identification	Peak intensity (Area)	Relative Percentage (%)
**1**	9.061	Unidentified	1.05E+07	0.98
**2**	9.406	α-pinene or 3-carene	1.53E+07	1.43
**3**	9.882	Camphene	8.95E+07	8.41
**4**	10.905	6-methyl-5-Hepten-2-one	2.30E+07	2.16
**5**	12.105	Limonene	2.04E+07	1.92
**6**	12.286	trans-β-Ocimene	1.02E+07	0.95
**7**	12.558	Unidentified	8.65E+06	0.81
**8**	13.156	4-Nonanone	1.47E+06	0.14
**9**	14.753	Decamethylcyclopentasiloxane (Siloxanes)	3.23E+07	**
**10**	16.948	**Citral**	3.06E+08	**28.77**
**11**	17.202	Geraniol	2.23E+07	2.09
**12**	17.556	**Citral**	3.62E+08	**33.94**
**13**	18.291	Dodecamethylcyclohexasiloxane	7.50E+07	**
**14**	19.707	Geranyl acetate	6.59E+07	6.18
**15**	20.633	Caryophyllene	8.18E+07	7.68
**16**	20.783	10,10-Dimethyl-2,6-dimethylenebicyclo[7.2.0]undecane	1.07E+07	1.00
**17**	21.288	Umulene	8.61E+06	0.81
**18**	21.416	Tetradecamethylcycloheptasiloxane	3.21E+07	3.01
**19**	22.291	1-Isopropyl-7-methyl-4-methylene-1,2,3,4,4*a*,5,6,8*a*-octahydronaphthalene	5.56E+07	5.22
**20**	22.366	[1*S*,*cis*]-Naphthalene,1,2,3,5,6,8*a*-hexahydro-4,7-dimethyl-1-[1-methylethyl]	8.52E+06	0.80
**21**	23.504	Caryophyllene oxide	1.65E+07	1.55
**22**	24.189	Hexadecamethylcyclooctasiloxane	1.15E+07	**
**23**	31.989	3,4'-Isopropylidenediphenol or 4,4'-Isopropylidenediphenol or 2,4'-Isopropylidenediphenol	2.89E+07	2.71

** Siloxanes release from the sampler and GC column.

Table S2. SPME-GC-MS peak identification and quantification in NO (examined at concentration of 1 mg/mL).

Entry	Retention Time (min)	Compound identification	Peak intensity (Area)	Relative Percentage (%)
**1**	5.027	Unidentified	5.42E+05	0.01
**2**	9.195	α-Pinene or α-Phellandrene	1.29E+07	0.26
**3**	9.401	α-Pinene	9.62E+08	19.12
**4**	10.675	β-Pinene	1.39E+08	2.76
**5**	11.042	β-Mircene	3.61E+07	0.72
**6**	11.774	4-Terpinenyl acetate	5.54E+06	0.11
**7**	11.965	*o*-Cymene or *m*-Cymene or *p*-Cymene	1.07E+08	2.13
**8**	12.096	Limonene	5.56E+08	11.05
**9**	12.103	**1,8-Cineole or Eucalyptol**	2.24E+09	**44.60**
**10**	12.848	4-carene or 3-carene	3.27E+07	0.65
**11**	13.531	4-methyl-3-(1-methylethylidene)-g-clohexene	2.14E+07	0.43
**12**	14.748	Decamethylcyclopentasiloxane	2.02E+07	**
**13**	16.051	α-Terpineol	6.36E+07	1.26
**14**	18.290	Dodecamethylcyclohexasiloxane	3.01E+07	**
**15**	19.155	α-Terpinyl formate	2.12E+07	0.42
**16**	20.637	Caryophyllene	5.45E+07	1.08
**17**	20.785	Unidentified	9.38E+06	0.19
**18**	21.377	Unidentified	2.40E+07	0.48
**19**	21.417	Tetradecamethylcycloheptasiloxane	1.39E+07	**
**20**	21.924	Unidentified	5.18E+07	1.03
**21**	23.026	Nerolidol	1.50E+08	2.98
**22**	23.310	Unidentified	1.29E+07	0.26
**23**	23.504	Unidentified	3.04E+07	0.60
**24**	23.714	[1*aR*-(1*a*α,4β ,4*a*β,7α,7*a*β,7*b*α)]-decahydro-1,1,4,7-tetramethyl-1*H*-cycloprop[*e*]azulen-4-ol	4.30E+08	8.54
**25**	23.889	Globulol	6.77E+07	1.35
**26**	24.186	Hexadecamethylcyclooctasiloxane	1.58E+07	**

** Siloxanes release from the sampler and GC column.

Table S3. SPME-GC-MS peak identification and quantification in ELO (examined at concentration of 1 mg/mL).

Entry	Retention Time (min)	Compound identification	Peak intensity (Area)	Relative Percentage (%)
**1**	8.483	Unidentified	1.02E+06	0.03
**2**	9.402	α-pinene	1.21E+09	31.51
**3**	10.681	β-pinene	2.12E+07	0.55
**4**	11.480	α-Phellandrene	1.23E+07	0.32
**5**	11.969	o-Cymene or m-Cymene or p-Cymene	8.67E+07	2.25
**6**	12.101	Limonene	2.18E+08	5.66
**7**	12.169	**1,8-Cineole or Eucalyptol**	2.06E+09	**53.52**
**8**	12.845	α-Phellandrene or γ-Terpinene	5.11E+06	0.13
**9**	14.751	Decamethylcyclopentasiloxane	1.72E+07	**
**10**	14.841	Unidentified	1.59E+06	0.04
**11**	15.328	Pinocarvone	6.97E+06	0.18
**12**	18.292	Dodecamethylcyclohexasiloxane	3.66E+07	**
**13**	19.160	α-Terpineol	1.67E+07	0.43
**14**	20.984	Aromandendrene	8.62E+07	2.24
**15**	21.378	Aromandendrene	1.60E+07	0.41
**16**	21.409	Tetradecamethylcycloheptasiloxane	1.10E+07	0.28
**17**	23.156	(-)-Globulol	1.02E+07	0.26
**18**	23.564	(-)-Globulol	4.09E+07	1.06
**19**	24.187	Hexadecamethylcyclooctasiloxane	1.98E+07	**
**20**	31.960	3,4'-Isopropylidenediphenol or 4,4'-Isopropylidenediphenol or 2,4'-Isopropylidenediphenol	5.36E+07	1.39

** Siloxanes release from the sampler and GC column.

Table S4. ATR-FTIR peaks' height and area (n = 3, S.D. < 3%).

Peaks		PCL	PCLaLGO	PCLaNO	PCLaELO	PCLbLGO	PCLbNO	PCLbELO
CH_2 symmetric	Wavenumber (cm^{-1})	2944	2944	2945	2946	2945	2945	2944
	Height (Abs)	0.042	0.060	0.076	0.080	0.084	0.066	0.074
	Area	1.690	2.463	3.132	3.296	4.190	2.743	3.093
CH_2 asymmetric	Wavenumber (cm^{-1})	2863	2865	2865	2865	2865	2865	2864
	Height (Abs)	0.021	0.032	0.039	0.042	0.037	0.034	0.038
	Area	0.426	0.621	0.756	0.793	0.726	0.673	0.710
C=O	Wavenumber (cm^{-1})	1721	1721	1721	1721	1721	1721	1721
	Height (Abs)	0.360	0.554	0.705	0.748	0.685	0.612	0.683
	Area	10.063	13.083	15.911	16.694	15.849	14.113	15.738
C–O, C–C crystalline	Wavenumber (cm^{-1})	1293	1294	1294	1294	1294	1294	1294
	Height (Abs)	0.058	0.086	0.099	0.104	0.091	0.083	0.092
	Area	0.765	1.117	1.308	1.395	1.190	1.101	1.216
C–O, C–C amorphous	Wavenumber (cm^{-1})	1162	1162	1161	1161	1161	1161	1161
	Height (Abs)	0.195	0.264	0.296	0.320	0.283	0.275	0.285
	Area	8.766	11.960	13.546	14.417	12.833	12.311	12.913
C–O–C asymmetric	Wavenumber (cm^{-1})	1239	1239	1239	1239	1240	1239	1239
	Height (Abs)	0.107	0.145	0.160	0.170	0.150	0.141	0.150
	Area	1.810	2.491	2.798	2.931	2.640	2.446	2.655

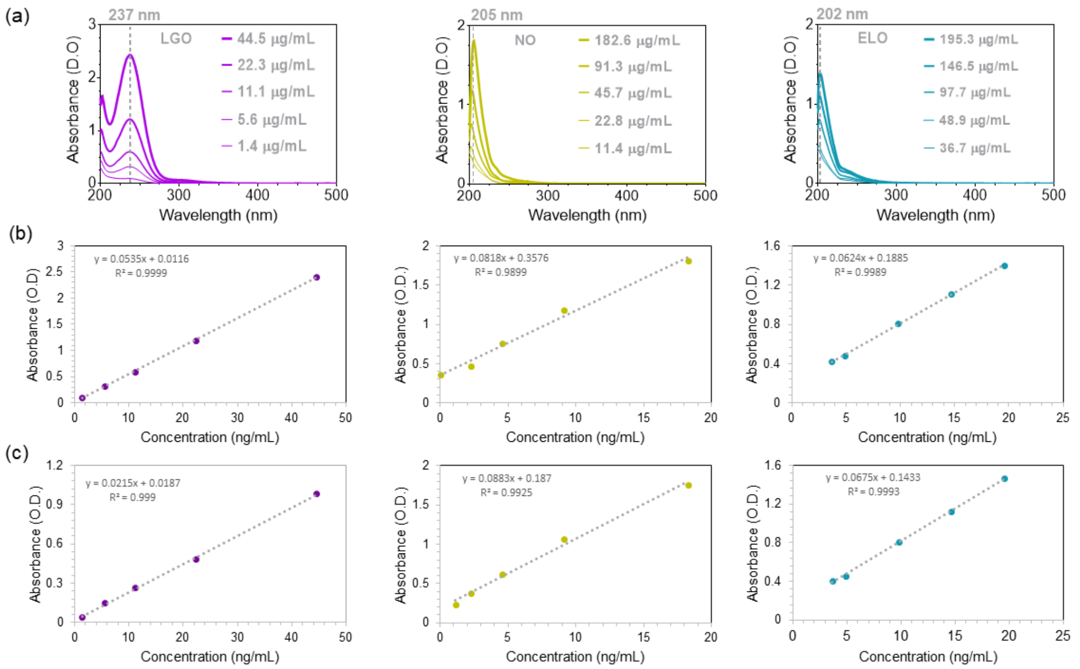

Figure S2. (**a**) Characteristic curves of LGO, NO and ELO at increasing EO concentration, determined using UV-visible spectroscopy. Calibration curves of LGO, NO and ELO extracted using (**b**) the UV-1800 and (**c**) the UV-2600.

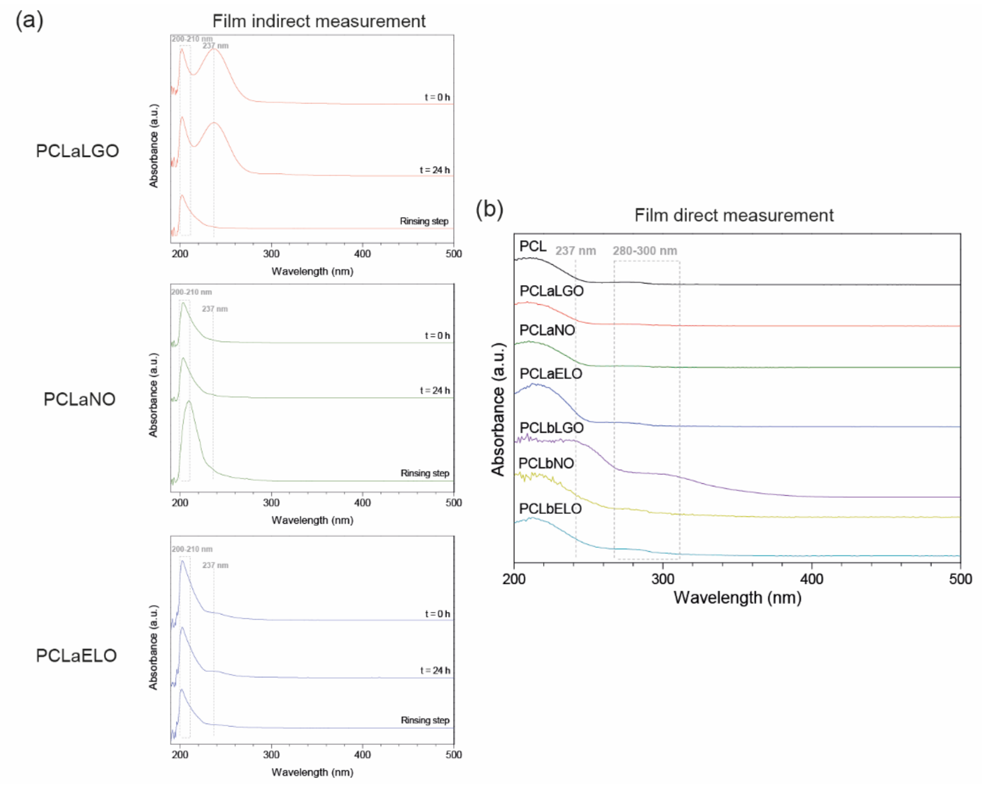

Figure S3. UV-visible absorption curves of representative (**a**) liquids used in the preparation of PCLaEOs mats, namely EO solution at t = 0 h of incubation with the PCL films, the same solution following 24 h of contact with the immersed films, and ethanolic rinsing solution after the incubation period. For each EO, an ideal dilution factor was considered for the solutions collected at t = 0 h and t = 24 h, namely of 1600× for LGO, 400 for NO and 600× for ELO. Rinsing solutions were all ran at a dilution of 10×. The 200–210 nm region comprehends characteristics peaks of NO and ELO. Wavelength of 237 nm is characteristic of citral (main LGO component). (**b**) Unloaded and loaded

PCL electrospun mats in the solid state. Shifts in the 280–300 nm region represent phenolic molecule rearrangements within the mats.

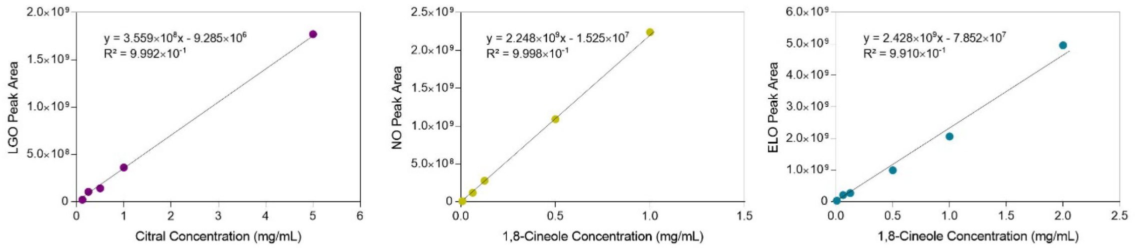

Figure S4. LGO, NO and ELO GC-MS calibration curves of the major components of each oil, namely citral and 1,8-cineole, tested for 4 h at 35 °C.

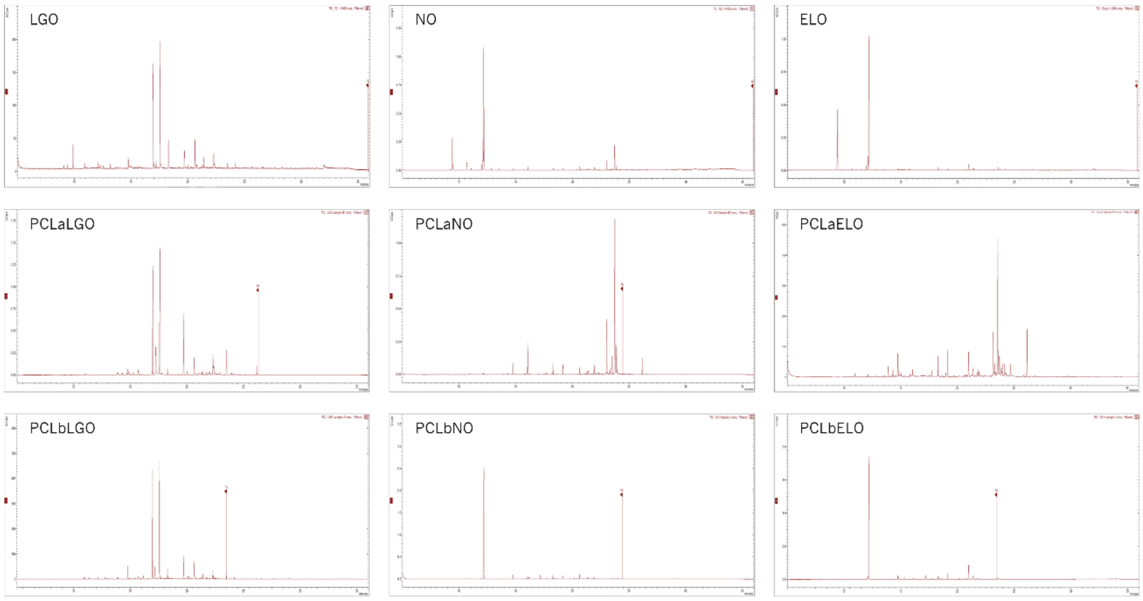

Figure S5. GC-MS spectra of the free EOs, LGO, NO and ELO prepared at 1 mg/mL concentration in ethanol, and the PCL mats absorbed and blended with the oils, PCLaEOs and PCLbEOs respectively.

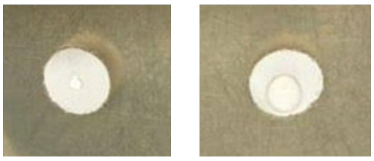

Figure S6. Photograph of a mat (exemplified with PCLbLGO) seeded with a bacteriophage droplet of (left) 5 µL and (right) 50 µL, for substrate and drop relative sizes comparison purposes. The first was used for permeability examinations (4 and 24 h at 37 °C), while the second was employed for contact inactivation studies (4 h at RT). After 4 h testing, the 5 µL droplet still remained on top of the surfaces.
